# Traditional Mediterranean Lifestyle: From a Heritage to a Legacy as an Inspirational Roadmap to a New Era^☆^

**DOI:** 10.1016/j.advnut.2026.100664

**Published:** 2026-05-30

**Authors:** Rena I Kosti, Ioanna Panagiota Kalafati, Matina Kouvari

**Affiliations:** Department of Nutrition and Dietetics, Nutritional Epidemiology and Communal Health Laboratory, School of Physical Education, Sports and Dietetics, University of Thessaly, Trikala, Greece

**Keywords:** Mediterranean diet, Mediterranean lifestyle, moderation, mindfulness, frugality, belongingness, purpose in life, sustainability, health

## Abstract

Grounded in the field of nutrition anthropology, the present manuscript introduces 3 core pillars of the traditional Mediterranean lifestyle: cheerful frugality, belongingness, and purpose in life. These foundational components are integral to Mediterranean culture and have historically shaped food selection, eating, and consumption patterns, depicting the harmonious human-nature-human coexistence. Specifically, the proposed core pillars highlight the role of alignment with natural rhythms in fostering cheerful frugality, the contribution of belongingness to social cohesion, and the influence of spirituality and religiosity in cultivating purpose in life. Together, these 3 pillars form a dynamic system that induces dispositional mindfulness, supporting and enhancing the salutogenic effect of the traditional Mediterranean lifestyle - beyond foods alone. Dispositional mindfulness emerges as the key tool that directs the interaction of these 3 pillars toward the traditional Mediterranean diet characteristic of moderation. Building upon this triptych and the crucial role of dispositional mindfulness, the manuscript proposes a roadmap to moderation, grounded in the ancient Greek concepts of *autarkeia* – self-sufficiency, and *enkrateia* – self-control. Within this perspective, *autarkeia* reframes frugality as cheerfulness, belongingness as meaningful connection, and purpose in life as a stable psychological resource. In this sense, mindfulness is suggested as a coping strategy for stressful situations with contemporary relevance for health-promoting lifestyles as the key tool for moderation.

**Statements of Significance**

This manuscript highlights the importance of mindfulness as a tool for moderation in contemporary diets and lifestyles. Emphasizing the crucial role of interconnectedness and harmony with social and natural environments, it proposes a roadmap to moderation that supports health and well-being.

## Introduction

The term “diet” derives from the ancient Greek word *diaita*, meaning “way of life” or “mode of living” [1]. In this sense, *diaita* encompassed not only what to eat, but also when to eat and how to live in a way that supports health and human flourishing. The traditional Mediterranean diet (tMedDiet) has been defined as “the diet prevailing in the Mediterranean basin up to the early 1960s, characterized by areas where olive trees grew on acres” [2]. This chronological threshold is important for defining what constitutes the traditional diet and for understanding which elements of Mediterranean cultural heritage were inscribed by United Nations Educational, Scientific and Cultural Organization (UNESCO) ∼15 y ago [3]. The UNESCO inscription recognizes not only a set of foods but also a broader cultural system that includes knowledge, practices, rituals, and social traditions related to food production, preparation, and consumption. Accordingly, the Mediterranean diet was recognized together with the skills, knowledge, symbols, and traditions associated with a lifestyle that was dominant in the region during that period [[1], [2], [3]]. Despite sharing core food groups with other healthy dietary patterns, the tMedDiet represents a highly diversified heritage expressed across different societies and social contexts [4,5]. Since its UNESCO inscription, the number of studies documenting the health benefits of tMedDiet on health outcomes has substantially increased [6]. Extensive evidence links adherence to this dietary pattern with reduced risk of cardiovascular disease, stroke, type 2 diabetes, certain cancers, cognitive decline, and all-cause mortality [6,7].

Simultaneously, the identification of additional cultural elements of tMedDiet - led to its transformation into the traditional Mediterranean lifestyle (tMedL), [[8], [9], [10], [11], [12]] described as a diverse and adaptable way of life encompassing conviviality, social connectedness, purpose in life, strong community and family bonds, harmony with nature, spirituality, religiosity, preservation of local traditions and resilience, underscoring moderation as its central virtue [12]. However, no researcher has attempted to outline the road to moderation as derived from the components of the tMedL so far.

Therefore, the aim of the present manuscript was to propose a conceptual perspective outlining a potential roadmap to moderation through a step-wise and integrative approach by introducing the notion of dispositional mindfulness. The concept of “mindfulness” or “state mindfulness” is rooted in Buddhist philosophy, and has been defined as “the awareness that arises from paying attention in a particular way: on purpose, in the present moment, and non-judgmentally” [13]. On the contrary, the often interchangeably used term of “dispositional mindfulness” or “trait mindfulness” refers to mindfulness as a stable individual characteristic that differs from person to person [13]. It is worth mentioning that, beyond genetic and developmental differences, dispositional mindfulness is influenced by environmental factors [14]. Thus, within the context of modern societal challenges—including unhealthy dietary patterns, stress, anxiety, social isolation, and depressive disorders, all of which have been associated with increased risk of all-cause mortality [15,16] — we propose that moderation, as shaped through dispositional mindfulness, may represent a transferable and enduring value. In light of emerging evidence supporting the role of balanced dietary patterns such as the tMedDiet in brain health [17], along with the fact that a meaningful and manageable orientation in life, defined as sense of coherence [18], is a core element of salutogenesis (“a resource-oriented approach to people’s abilities”) [19], we discuss how a mindful lifestyle may contribute to salutogenesis. In doing so, this perspective seeks to complement existing evidence by offering a broader, integrative framework that highlights the potential added value of the Mediterranean lifestyle beyond its nutritional components alone.

In this study, first, we conceptualize how dispositional mindfulness may emerge from key components of the tMedL by presenting its core pillars and examining their potential interactions. Second, we discuss possible intermediary mechanisms that may contribute to moderation, drawing on the ancient Greek concepts of *autarkeia* (self-sufficiency) and *enkrateia* (self-control).

## The Core Pillars of the tMedL

**FIGURE 1:**
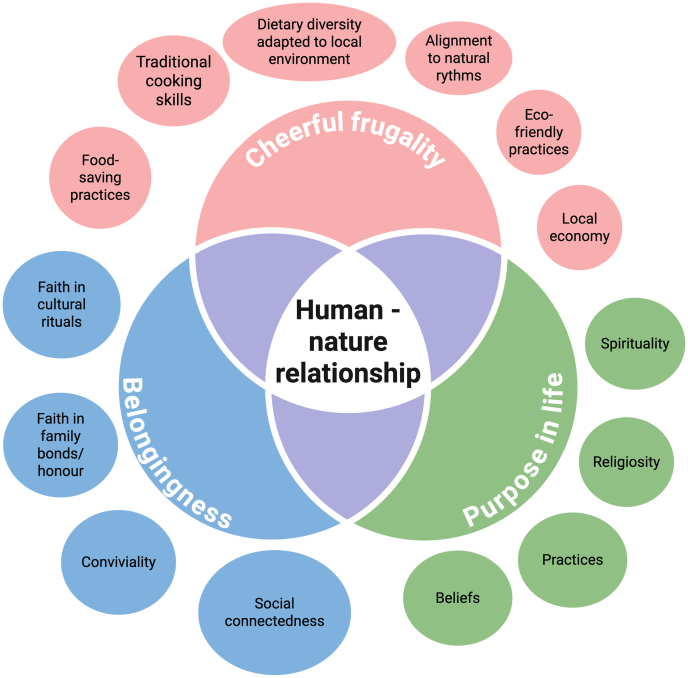
The core pillars of tMedL philosophy. Created in BioRender (by IPK, 2026). tMedL: traditional Mediterranean lifestyle.

**FIGURE 2:**
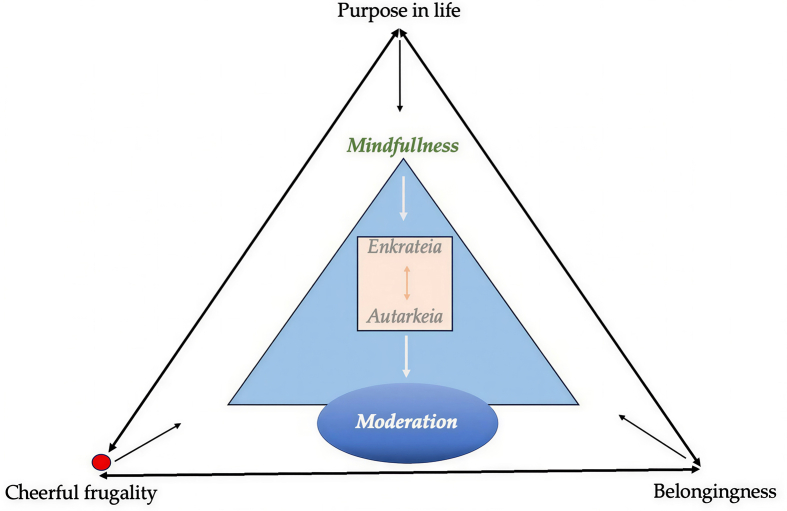
The roadmap to moderation.

In this manuscript, we propose a conceptual framework to describe how key components of the tMedL may contribute to moderation through dispositional mindfulness. Within this framework, 3 core pillars are identified: *1*) cheerful frugality, *2*) belongingness, and *3*) purpose in life - whereas the roadmap refers to the step-wise and dynamic interactions through which these pillars may influence behavior and health outcomes (FIGURE 1, FIGURE 2).

The three proposed pillars are presented in a sequential and hierarchical manner. Cheerful frugality is positioned as the foundational principle, reflecting a harmonious relationship between humans and the natural environment. From this foundation emerges belongingness, expressed through social connectedness and shared practices, which in turn supports the development of a deeper sense of purpose in life. This sequence is supported by evidence depicting all the elements, foods, habits, values, and principles of the tMedL linking Mediterranean lifestyle patterns with well-being and longevity [[8], [9], [10], [11], [12],^20^] (Figure 1).

In tMedL, it all starts from the natural environment, landscapes, the know-how of ancient agricultural practices, cultural, social, and food traditions [21]. Originally, the Mediterranean diet was characterized by the “bread-olive oil-wine” triad, enriched with legumes and fermented dairy products originating from lean sheep and goats. Meat was consumed occasionally, such as during religious festivals or weddings [21].

### Cheerful frugality

Cheerful frugality, as the foundational pillar of the tMedL, reflects a way of living characterized by moderation, resourcefulness, and alignment with natural cycles. The tMedL has been promoted by emphasizing frugality that is fundamental to it, as a traditionally “food-saving culture” [9]. Indeed, it seems that frugality and sustainability are closely intertwined through tMedL practices such as dietary diversity adapted to the local environment and economy, biodiversity, seasonality, eco-friendly family farming, minimal levels of food processing, food-saving habits, simplicity, and the preservation of traditional cooking skills [11,12]. The foundation of the tMedL is the moderate, frugal, and prudent consumption of a variety of mainly plant-based foods, which is crucial to the health advantages this diet is thought to offer [9,10]. Respect for natural resources and their seasonal rotation is crucial in tMedL. This tMedL characteristic is consistent with the environmental aspect of well-being, which embraces the peaceful coexistence of humans and the natural world [10,12]. Literature supports the view that frugal behavior is significantly and beneficially influenced by the 3Ds of consciousness for sustainable consumption, which are economic, environmental, and social, as well as by consideration of long-term future consequences, promoting a moderate consumption of resources [22]. In this sense, frugal behavior can be viewed as a combination of self-controlled actions based on limited, regulated consumption and creative use of available resources [23]. In this regard, it is not coincidental that the traditional Cretan diet, which is the reference diet of the tMedDiet, was characterized by profound ancestral knowledge of the land – including what it offers, when and where resources can be found, and how they can be prepared. The proverb *“When the donkey starves, the Cretan gets fat”* reflects this practical wisdom: although the donkey consumes only the upper leaves of a plant, its owner may also utilize its roots, symbolizing ingenuity and resourcefulness in the use of available foods. A reasonable question that may arise is why the term “cheerful frugality” is preferred rather than “frugality,” particularly when the tMedDiet was historically been described a “diet of poverty” due to necessity rather than choice [24]. If the tMedDiet was only a “diet of poverty,” it would be difficult to explain why descriptions of this dietary pattern consistently emphasize that family and communal meals are characterized by social interaction, communication, conviviality, warmth, and friendliness – the pleasure derived from meal sharing – as fundamental elements of the region’s food culture and daily life [8,[25], [26], [27]]. Eating with family and friends promotes slow, communal, and pleasurable eating, fostering intergenerational bonds, reinforcing a sense of belonging within the community, and reducing stress [25,28]. The well-known phrase often attributed to the Greek philosopher and historian Plutarch, “We do not sit at table to eat but to eat together,” highlights the concept of commensality [26].

Moreover, conviviality, defined as “the quality of being friendly and lively” and commensality – defined as “the act of eating together” [28]- extend beyond their social impact, acting as a regulating force in food intake, thereby encouraging mindful eating as well as beneficial effects on health [26,[28], [29], [30]]. Moreover, literature suggests that tMedL components such as communal or field-based activities, entailing physical activity and sun exposure, together with post-lunch siestas, promote circadian rhythms that are more closely aligned with natural cycles, leading to improved sleep quality, enhanced happiness, and better health [[31], [32], [33], [34], [35]]. Alignment with natural rhythms through physical activity and adequate sleep has also been associated with mindfulness, which in turn enhances well-being and supports a more balanced lifestyle [36,37]. Research supports the view that individuals who prioritize simplicity in life display enhanced positive psychology and well-being [38]. Together, simplicity, frugality, and purposeful living form a coherent approach to navigating life’s complexity by preserving focus on the values that sustain genuine happiness [39]. Resilience in the face of adversity was described as one of the components of tMedL [12]. Moreover, resilience and mindfulness are closely intertwined, dynamic processes of adaptation to adversity; meta-analytic findings confirmed that individuals with higher levels of dispositional mindfulness seem to have higher levels of resilience [40]. The simplicity of lifestyle and a distance from abundance achieved through the conservation of natural resources open the mind to spirituality, defining frugality as “art de vivre” [41].

### Belongingness

Belongingness is defined as the personal sense of a profound connection to social groups, physical environments, and both individual and shared experiences [42]. As illustrated above, frugality can also strengthen human connectedness, a core tMedL component incorporating the notion of “belongingness” [12], herein suggested as the second pillar of the tMedL philosophy. A recently published umbrella review confirmed a positive association between strong social bonds and health outcomes, highlighting how features such as family ties and orientation, conviviality, faith in cultural rituals, and interdependence – common in Mediterranean culture – are beneficial for longevity and well-being, particularly in older adults [43,44]. In contrast, social isolation and loneliness have been associated with 32% and 14% higher mortality risks, respectively [45]. Furthermore, social exchange theory highlights the role of reciprocity, trust, and shared practices – often shaped by frugality – in fostering prosocial behavior [46]. Prosocial behaviors include any activity aiming to benefit others or society, such as sharing, volunteering, and cooperating, and are essential for social functioning [47]. Indeed, the social fabric of Mediterranean societies was shaped by spiritual and religious communities that exhibited prosocial behaviors and fostered a sense of belonging, thereby contributing to community cohesion [12,48]. It is worth mentioning that prosocial behaviors are positively associated with mindfulness [49] and nature exposure [50], providing health-promoting benefits. A related form of belonging, nature connectedness, is associated with well-being and purpose in life [51]. Moreover, research shows a strong connection between belonging and experiencing purpose in life[52].

### Purpose in life

Purpose in life, suggested herein as the third core pillar of the tMedL, along with belonginess, pertains to an individual’s intrinsic need to experience a deep sense of connection and belonging either to the wider natural environment or to the broader human community, respectively [53]. These constructs can be viewed as complementary dimensions of the same underlying condition, each exerting distinct effects on mental health and mindfulness [53]. Purpose in life, as expressed through spirituality, religiosity, beliefs, and practices in tMedL [12], can act as a means of coping with stressful life situations, since it has been found to reduce the effects of depression and anxiety [54]. The tMedL can be linked with spirituality and religion through its connection to nature, encompassing a broader intersection of dietary patterns and ethical/spiritual practices that emphasize well-being, community, and reverence for natural and symbolic elements of food [4]. Purpose in life has also been associated with reduced all-cause mortality regardless of gender and race/ethnicity [55,56]. The available research shows that more spiritual people tend to have lower rates of chronic illness and lower mortality risk, although direct causal evidence is not yet confirmed due to a complex relationship [57]. Both engagement in activities and a sense of purpose in life are linked to mindfulness, and both are linked to favorable health outcomes [58].

### The roadmap to moderation

It is worth noting that the 3 proposed pillars do not operate independently. Rather, they function sequentially, synergistically, and in full coordination, within a dynamic loop inducing salutogenesis through mindfulness. Notably, “purpose in life” appears to be strengthened and ultimately culminates through the synergistic effects of “cheerful frugality” and “belongingness,” implying that the human-nature-human relationship serves as the nuclear link of the 3 pillars [51,52,58,59]. Together, these 3 pillars form a coherent pathway toward mindful living [58,[60], [61], [62]] (Figure 2). The interactions are bidirectional, given the fact that mindfulness has been found to enhance purpose in life by attenuating self-boundaries [62].

## Dispositional Mindfulness as a Tool for Moderation

Mindfulness has been shown to promote life satisfaction [63] through the moderating role of self- control [64], which in turn has been identified to be a predictor of longevity [65,66], a well-documented attribute of the tMedDiet. Moreover, it appears that dispositional mindfulness is also associated with self-regulation intertwined with self-control [67], which in turn leads to conscious choices that contribute to a healthy lifestyle [68], encompassing a balanced diet, regular physical activity, quality sleep, and the formation of strong social bonds within the frame of a sustainable natural environment. In ancient Greek philosophy, self-control (*enkrateia*) [69] is the ability to regulate one’s own desires and inclinations [70]. Self-control is closely linked with self-sufficiency (*autarkeia*) as it enables true independence and supports a flourishing life [71,72]. *Autarkeia* does not imply the rejection of material goods, but rather a state in which life is complete and lacking nothing. Within this framework, moderation is the outward expression of a well-controlled and self-sufficient individual [73] (Figure 2).

## From a Heritage to a Legacy

The triptych of the 3 pillars as core values of the tMedL is the crossroads of ancient Greek philosophy and contemporary lifestyles. It could sound provocative, yet one could presume that these values could “feed” the degrowth movement. The degrowth movement employs the concept of “frugal abundance” to describe a mode of living that prioritizes simplicity while remaining rich in cultural, emotional, and spiritual dimensions [74]. Within this framework, individuals’ basic needs are met collectively and sustainably in ways that honor the ecological limits of the planet. This contemporary approach to “frugal abundance” resembles the core pillar of “cheerful frugality” of the tMedL. Even after 5000 y, the quote of Aristotle “Metron Ariston,” implying “nothing in excess and nothing in deficiency” – not as a strict average, but as a context-specific, rational judgment – remains profoundly timeless [75]. Considering the contradicting and in some cases even misleading outcomes observed in nutrition and health today – ranging from metabolic disorders to mental health challenges – can be traced to a fundamental deficit: the erosion of moderation in daily life. By embedding mindfulness, self-sufficiency, and a harmonious connection with both human and natural environments, the tMedL offers a blueprint of resilience, longevity, and holistic well-being. The acquisition of the cognitive skill of mindfulness through deliberate practice, or the enhancement of dispositional mindfulness through human-nature interactions as a means to achieve moderation, could facilitate the adoption of a lifestyle more resilient to stress, in favor of human well-being and sustainable food consumption [76]; a challenging endeavor, but one well worth pursuing.

## Author contributions

The authors’ responsibilities were as follows – RIK: wrote the paper and had primary responsibility for the final content; IPK: prepared the figures; RIK, IPK, MK: edited the manuscript and critically reviewed it; and all authors: read and approved the final manuscript.

## Declaration of AI and AI-Assisted Technologies in the Writing Process

The authors declare that no generative AI or AI-assisted technologies were used in the writing of this manuscript.

## Funding

Nothing to declare.

## Conflict of interest

The authors report no conflicts of interest.
